# Work engagement, well-being, and intent to continue working based on educational support among foreign care workers in Japan

**DOI:** 10.1265/ehpm.21-00248

**Published:** 2022-02-19

**Authors:** Junko Kameyama, Yumi Hashizume, Yuko Takamura, Shoko Nomura, Tomoki Gomi, Hisako Yanagi

**Affiliations:** 1 Research Fellow, Department of Neurosurgery, Faculty of Medicine, University of Tsukuba, Ibaraki, Japan; 2 Department of Health Innovation and Nursing, Faculty of Medicine, University of Tsukuba, Ibaraki, Japan; 3 Department of Nursing, School of Health Sciences Ibaraki Prefectural University of Health Science, Ibaraki, Japan; 4 Graduate School of Comprehensive Human Sciences Master’s Program in Public Health, University of Tsukuba, Ibaraki, Japan

**Keywords:** Human resource development, Well-being, Work engagement, Foreign workers, Long-term care, Practical educational support

## Abstract

**Background:**

Global aging continues to progress. The shortage of human resources involved in long-term care (LTC) is a serious problem worldwide. It is necessary to promote the stable employment of foreign care workers. The purpose of this study was to identify which factors, including well-being, work engagement, and original items, contribute to foreign care workers’ intent to continue working.

**Methods:**

We conducted an anonymous self-administered questionnaire survey of 259 foreign LTC workers at LTC facilities in Japan. The questionnaire survey items included the Japanese version of the Subjective Well-being Scale (J-SWBS), the Japanese version of the Utrecht Work Engagement Scale (J-UWES), and original items related to educational needs and issues. We used multiple regression analysis to predict variability from correlations among variables. And after that, we conducted a path analysis using structural equation modeling (SEM), and added that the explanatory variables (IV) were well-being, work engagement, and the original item component, and that the outcome variable (DV) was intention to continue working. We set a hypothetical model based on structural equations, corrected by path analysis, and examined its suitability.

**Results:**

The number of returned questionnaires for 259 foreign care workers was 147 (response rate 56.7%), and the number of analyzable questionnaires was 129 (valid response rate 49.8%). For intention to continue working, the results of structural equation modeling showed direct effects for satisfaction with low back pain measure guidance (β = .255), satisfaction with the national examination guidance method (β = .217), well-being (β = .046), and work engagement (β = .026). In work-engagement, there was a direct effect of happiness (β = .715), willingness to learn good care (β = 4.849), and confidence in my ability (β = 2.902,), whilst in well-being, satisfaction with low back pain measure guidance (β = 1.582) and confidence in my ability (β = 1.999) were found to have direct effects.

**Conclusions:**

To increase the intent of foreign care workers to continue working, appropriate guidance should be given related to the development of lumbago. In addition, to provide a place and scene where they can learn good care, having a relationship in practice where foreign care workers can feel that their abilities are being utilized, and developing and maintaining educational support that motivates them to learn good care may be effective.

## Background

The estimated demand for LTC personnel in Japan in fiscal year 2025 is 2.53 million. On the other hand, the estimated supply, which reflects the projected decrease in the working-age population in the future, is 2,152,000, resulting in a shortage of 377,000 workers [1]. This is an estimate that requires an increase of approximately 60,000 workers per year. However, in the care and welfare sector, where the turnover rate of LTC workers has been approximately 15% to 21% since 2007 [2, 3], and the turnover rate in all industries including health care and welfare is in the 14% to 17% range [4], the annual increase in the number of LTC workers will be about 40,000. On the other hand, the number of foreign workers in the medical and welfare fields as of 2020 has increased 2.5 times compared to 2016 [5]. However, the retention rate in long-term care (LTC) facilities of care worker candidates (candidates), who are the leaders in the field of LTC workers, is only about 14% of the total number of entrants [6].

Previous studies have identified a number of factors related to turnover of staff involved in older care, such as poor quality of older care [7], occupational stress [7, 8], decreased positive attitude toward work [8], lack of technical guidance, high demands from the workplace, and poor relationship with superiors [8, 9]. However, most of the literature published so far is based on studies of native workers. Although recruitment is the most important issue in the field of LTC, specific support methods focusing on the retention of foreign LTC workers remain unclear. At present, there is no research that presents a theory of educational support methods for the purpose of retaining foreign care workers.

The purpose of this study was to identify which of the original items extracted based on the results of our own previous study contributed to well-being and work engagement, as well as to the intention of foreign LTC workers to continue working. Promoting the stable employment of foreign LTC workers will help to solve the problem of human resource shortage in the field of LTC for the older adults. This study focuses on securing LTC human resources in a comprehensive manner, including securing and training diverse human resources, preventing turnover, promoting retention, improving productivity, and improving the environment for accepting foreign human resources. We focused on the recruitment and development of diverse human resources, prevention of turnover, promotion of retention, improvement of productivity, and improvement of the environment for accepting foreign human resources. Solving the shortage of human resources is thought to lead to enhancement of employee welfare and improvement of physical and psychological health, and this study targeting foreign nationals has implications for transnational health.

## Methods

### Recruitment

The survey included 223 facilities that accept candidates based on the Economic Partnership Agreement (EPA), whose names have been disclosed according to the survey by the Ministry of Health, Labor and Welfare, as well as 69 facilities that disclose the acceptance of foreign workers on their websites. We used Microsoft Bing (https://www.bing.com) to search for the keywords “foreign human resources,” “long-term care,” and “receiving facilities.” The description for website search has been modified.

We explained the research outline in writing to these 292 facilities or corporations and requested their cooperation in the research. Of these, the foreign workers of 36 facilities that provided consent to accepting a mailed request form for this study were surveyed by mail through the intermediary of the person in charge of the facility.

### Inclusion criteria

The target participants were foreign LTC workers who had been working at LTC facilities in Japan for 6 months or more. Those with level N3 of the Japanese Language Proficiency Test (basic proficiency with everyday Japanese.) or those with equivalent or higher proficiencies were selected. There are five levels of the Japanese Language Proficiency Test: N1, N2, N3, N4, and N5. The easiest level is N5, and the most difficult is N1. N5 is the level at which you can understand basic Japanese to some extent, and N1 is the level at which you can understand Japanese used in various situations.

### Ethical considerations

For foreign care workers, the text of the request form was simplified into easy Japanese. All the survey forms were accompanied by a request form explaining the study’s purpose, methodology, ethical considerations, and so forth. The form also contained a statement that the respondent’s participation in the study was wholly voluntary, and after completing the survey, the participants themselves placed the questionnaire into the provided envelope and sealed it carefully.

This study was conducted with the approval of the University of Tsukuba medical ethics review committee (ethics approval number 1438).

### Composition of the questionnaire

#### 1) Demographics

The participants filled in questions about their nationality, biological sex, age, Japanese Language proficiency, years of work experience (months) in Japan, qualifications, and residency status.

#### 2) Intent to continue working

This item was “I want to continue working at my current workplace.” The response options were “Strongly agree,” “Agree,” “Neutral,” “Disagree,” and “Strongly disagree.”

#### 3) Well-being

The Japanese version of the Subjective Well-being Scale (J-SWBS) [10, 11] was used as the scale for measuring well-being. The SWBS is a “mental health self-assessment questionnaire” developed by the World Health Organization (WHO) to measure psychological health [10]. It consists of the following items: “positive feelings towards life” (3 items), “confidence” (3 items), “achievement” (3 items), “disappointment in life” (3 items for reversal), and “bliss” (3 items). There are different answer options according to the question content of each item, and each item is rated using a 4-point Likert scale. The overall scale scores range from 12 to 48 points. The higher the score, the higher the individual’s subjective psychological health [10].

#### 4) Work engagement

To measure work engagement, a Japanese version of the Utrecht Work Engagement Scale (J-UWES) [12, 13] was used. The UWES was developed by Schaufeli et al. Work engagement is a term that refers to the state of well-being related to work and was proposed as a counter-concept for burnout [12, 14]. Employees who are burned out are exhausted and their enthusiasm for work is reduced, whereas employees with high UWES are full of vitality and are actively involved in work [15, 16]. The overall scale scores range from 0 to 54 points: the higher the score, the higher the individual’s pride, enthusiasm, and energy and vibrancy toward the work [17].

#### 5) Original items

Nineteen items were created on the basis of the results obtained from a previous study [18]. The purpose of that study was similar to that of the current study; ie, it explored the factors that contribute to better educational support methods for foreign care workers to remain in the workforce. The instruction was “Please select one number (1 to 5) that describes the feeling that is closest to yours and write it on the mark sheet.” The choices differed according to the content of the items, and all the answers to the 19 items were obtained using a 5-point Likert scale.

### Outcomes

The primary endpoints were intent to continue working, well-being, work engagement, and original items. and the secondary endpoints were basic attributes.

### Statistical analyses

The Mann-Whitney U test was used to test for differences among the residency statuses of participants in well-being, and the Kruskal-Wallis test was used to test for differences among the nationalities of participants in work engagement. We used the Spearman rank correlation to analyze the correlation between the ordinal scale and the proportional scale for “intent to continue working” and “demographics,” SWBS, UWES, and “original items.” Also, referring to the analysis results, variables showing significant correlation with the SWBS, UWES, “demographics,” and “original items” were defined as independent variables. Multiple regression analysis was performed by inputting the variables by the forced input method, with “intent to continue working” as the dependent variable. Next, multiple regression analysis was conducted, where work engagement and well-being, which have high internal correlation, were analyzed separately. Path analysis using structural equation modeling (SEM) was conducted to analyze the hypothetical model created based on the variables that were found to be significant in the multiple regression analysis. The reliability of the original items in the questionnaire used in this study was verified by use of the Cronbach α coefficient.

Probability values below .05 (both sides) were considered to indicate significance. The statistical software used was SPSS Statistics, ver. 28 and Amos, ver. 27 (IBM).

## Results

Of the 259 foreign workers, 147 responded (response rate: 56.7%). Fifteen people whose number of years (months) worked at LTC facilities or whose Japanese proficiency did not meet the study criteria were excluded. Of the 132 people who met the inclusion criteria, 129 people were analyzed (valid response rate: 49.8%), excluding 2 missing answers and 1 incorrect answer such as duplicate answers.

### Demographics

Table 1 shows the statistics of the participants’ demographics. In terms of nationality, Vietnamese nationality was the most frequent, with 46 (35.7%), followed by Indonesian nationality, with 41 (31.8%), and Philippine nationality, with 32 (24.8%). The sample also included Chinese and Thai nationalities, but the number was very small (1 or 2). The median age was 28 years (range 20–58 years), and 102 of the respondents (85%) were female. Regarding the qualifications held at the time of the survey, nursing (75; 58.1%) and care work (29; 22.5%) qualifications accounted for 80% of the total. The care worker and care manager qualifications shown in the table are national qualifications acquired in Japan. Regarding their residency status, 99 people (76.7%) were qualified for residency on the basis of specific activities, that is, on the basis on the residency status set out in the EPA. In terms of length of employment in Japan, the largest number of respondents (54; 41.8%) had been working in Japan for more than 3 years but less than 5 years.

**Table 1:** Participants’ demographics

	n = 129

**Age** (years)	
Median	28
Interquartile range	26–30
Range	20–58
**Women** n (% ^†^)	102(85.0)^*^
Country of Citizenship n (% ^†^)	
Indonesia	41(31.8)
Vietnam	46(35.7)
Philippine	32(24.8)
China	2(1.6)
Thailand	1(0.8)
Others	7(5.4)
**Qualification** n (% ^†^)	
Nurse	75(58.1)
Certified caregiver	5(3.9)
Care worker ^‡^	29(22.5)
Care manager ^‡^	1(0.8)
Others	19(14.7)
**Status of residency** n (% ^†^)	
Specific activity	99(76.7)
Study abroad	2(1.6)
Technical intern training	15(11.6)
Specific skill	1(0.8)
Others	12(9.3)
**Working period in Japan** n (% ^†^)	
6 months to less than 1 year	16(12.4)
1 year to less than 3 years	39(30.2)
3 years to less than 5 years	54(41.8)
5 years to less than 10 years	15(11.6)
More than 10 years	8(6.2)

^*^: Calculated excluding unknown seven people from the total number

^†^: Rounded to the second decimal place

^‡^: Qualification in Japan

### Correlation between intent to continue working and various factors

Table 2 shows the correlation between the intent to continue working and participants’ demographics, work engagement, well-being, and original items. The original items from personnel sufficiency to damage frequency of verbal abuse or violence in this table were created on the basis of the characteristics categories of a previous study [18]. Items that showed a positive correlation with the intent to continue working were work engagement (r = .496, p < .01), well-being (r = .448, p < .01), ease of consultation with Japanese staff (r = .403, p < .01), sense of being valued (r = .510, p < .01), satisfaction with the number of holidays (r = .422, p < .01), salary satisfaction (r = .412, p < .01), satisfaction with the national examination guidance methods (r = .484, p < .01), sense of learning good care (r = .412, p < .01), confidence in my ability (r = .417, p < .01), satisfaction with low back pain measures guidance (r = .423, p < .01), and Japanese understanding of your own culture (r = .420, p < .01). The items that showed a weak correlation with the intent to continue working were personnel sufficiency (r = .297, p < .01), satisfaction with study time (r = .357, p < .01), pressure of the national exam (r = .201, p < .05), willingness to learn for good care (r = .352, p < .01), and family support (r = .359, p < .01). On the other hand, the item that showed a weak negative correlation to continue working was working period in Japan (r = −.232, p < .01).

**Table 2:** Correlation of Participants’ demographics and original items with intent to continue working, well-being, and work engagement

	**Intent to continue working**	**Well-being**	**Work engagement**	**Subscale**		

				**Vitality**	**Enthusiasm**	**Immersion**
Intent to continue working		.448^*^^*^	.496^*^^*^	.426^*^^*^	.432^*^^*^	.466^*^^*^
Age	−0.024	−0.058	−0.133	−0.099	−0.147	−0.074
Japanese language ability ^†^	0.148	0.154	.266^*^^*^	.219^*^	.322^*^^*^	.203^*^
Working period in Japan	−.232^*^^*^	−.199^*^	−.293^*^^*^	−.254^*^^*^	−.294^*^^*^	−.213^*^
Personnel sufficiency	.297^*^^*^	0.128	.250^*^^*^	.218^*^	.257^*^^*^	.259^*^^*^
Ease of consultation to Japanese staff	.403^*^^*^	.369^*^^*^	.271^*^^*^	.192^*^	.258^*^^*^	.249^*^^*^
Feeling of being cherished	.510^*^^*^	.477^*^^*^	.354^*^^*^	.340^*^^*^	.313^*^^*^	.330^*^^*^
Satisfaction with the number of holidays	.422^*^^*^	.255^*^^*^	.369^*^^*^	.339^*^^*^	.317^*^^*^	.384^*^^*^
Salary satisfaction	.412^*^^*^	.292^*^^*^	.434^*^^*^	.368^*^^*^	.387^*^^*^	.431^*^^*^
Number of night shifts ^†^	.175^*^	0.141	0.163	0.133	.224^*^	0.096
Satisfaction with the number of night shifts	0.093	0.062	0.136	0.149	0.092	0.158
Satisfaction with study time	.357^*^^*^	.318^*^^*^	0.173	0.079	0.149	.215^*^
Satisfaction with the national examination guidance methods	.484^*^^*^	.298^*^^*^	.267^*^^*^	.228^*^	0.183	.276^*^^*^
Pressure of the national exam	.201^*^	−0.057	0.052	0.026	0.012	0.101
Sense of learning good care	.412^*^^*^	.305^*^^*^	.358^*^^*^	.268^*^^*^	.351^*^^*^	.378^*^^*^
Sense of performing good care	.177^*^	0.155	.341^*^^*^	.268^*^^*^	.351^*^^*^	.378^*^^*^
Willingness to learn for good care	.352^*^^*^	.292^*^^*^	.506^*^^*^	.432^*^^*^	.528^*^^*^	.434^*^^*^
Confidence in my ability	.417^*^^*^	.358^*^^*^	.442^*^^*^	.408^*^^*^	.422^*^^*^	.370^*^^*^
Comfort of the conversation with the users	0.093	.282^*^^*^	0.035	−0.021	0.067	0.058
Satisfaction with low back pain measure guidance	.423^*^^*^	.333^*^^*^	.297^*^^*^	.239^*^^*^	.250^*^^*^	.318^*^^*^
Japanese understanding of my own culture	.420^*^^*^	.325^*^^*^	.283^*^^*^	.235^*^^*^	.283^*^^*^	.290^*^^*^
Family support	.359^*^^*^	.318^*^^*^	.225^*^	0.118	.250^*^^*^	.258^*^^*^
Damage frequency of verbal abuse, violence	0.031	0.054	0.02	0.085	−0.003	−0.052

Spearman rank correlation analysis   ^*^^*^p < 0.01 ^*^p < 0.05

^†^: Reversal item

### Correlation between work engagement, well-being, and various factors

As shown in Table 2, the items that showed a positive correlation with work engagement were well-being (r = .421, p < .01), salary satisfaction (r = .434, p < .01), willingness to learn for good care (r = .506, p < .01), and confidence in my ability (r = .442, p < .01). In addition, Japanese language ability (r = .266, p < .01), personnel sufficiency (r = .250, p < .01), ease of consultation with Japanese staff (r = .271, p < .01), sense of being valued (r = .354, p < .01), satisfaction with the number of holidays (r = .369, p < .01), satisfaction with the national examination guidance methods for care workers (r = .267, p < .01), sense of learning good care (r = .358, p < .01), sense of performing good care (r = .341, p < .01), satisfaction with low back pain countermeasure guidance (r = .297, p < .01), Japanese understanding of your own culture (r = .283, p < .01), and family support (r = .225, p < .05) showed a weak correlation with work engagement. Whereas working period in Japan (r = −.293, p < .01) showed a weak negative correlation with the intent to continue working, satisfaction with training (study) time and pressure of the national exam did not correlate with the intent to continue working.

Well-being had a positive correlation with sense of being valued (r = .477, p < .01), and comfort of conversation with users in Japanese (r = .292, p < .01) had a weak correlation that was not seen between the intent to continue working and work engagement.

### Factors affecting the intent to continue working

Tables 3 and 4 show the results of multiple regression analysis with intention to continue working as the dependent variable. In Table 3, the following 5 factors were independently related to the intent of foreign care workers to continue working (r^2^ = .590, p < .001): work engagement (β = .314, p = .041), satisfaction with the national examination guidance method (β = .188, p = .047), and sense of performing good care (β = −.233, p = .020), confidence in my ability (β = .212, p = .029), and satisfaction with low back pain measure guidance (β = .239, p = .011). In Table 4, the following 4 factors were independently associated (r^2^ = .561, p < .001): well-being (β = .210, p = .041), willingness to learn good care (β = .193, p = .044), confidence in my ability (β = .246, p = .014), and satisfaction with low back pain measure guidance (β = .215, p = .029).

**Table 3:** Factors that affect the intent to continue working

	***β***	**t value**	***P* value**

Work engagement	0.314	3.218	0.002
Japanese language ability ^†^	−0.067	−0.799	0.427
Working period in Japan	−0.022	−0.252	0.801
Feeling of being cherished	0.161	1.48	0.143
Salary satisfaction	−0.045	−0.423	0.674
Satisfaction with study time	−0.04	−0.465	0.643
Satisfaction with the national examination guidance method	0.188	2.017	0.047
Pressure of the national exam	0.051	0.686	0.495
Sense of learning good care	0.025	0.23	0.818
Sense of performing good care	−0.233	−2.375	0.020
Willingness to learn for good care	0.166	1.807	0.074
Confidence in my ability	0.212	2.229	0.029
Satisfaction with low back pain measure guidance	0.239	2.601	0.011
Japanese understanding of my own culture	0.017	0.187	0.852

Dependent variable; Intent to continue working. *P* value; Multiple regression analysis. Forced input method

Model summary: R = 0.768, R^2^ = 0.59, adjusted R^2^ = 0.519, standard error of estimates = 0.635

^†^: Reversal item: N1 ∼ N5 (Lower numerical value N1 is the highest level)

Note; The independent variables in Table 3 and Table 4 are the same except for “work engagement” and “well-being”.

**Table 4:** Factors that affect the intent to continue working

	***β***	**t value**	***P* value**

Well-being	0.21	2.081	0.041
Japanese language ability ^†^	−0.032	−0.364	0.717
Working period in Japan	−0.004	−0.04	0.968
Feeling of being cherished	0.173	1.531	0.13
Salary satisfaction	0.03	0.272	0.786
Satisfaction with study time	−0.068	−0.739	0.462
Satisfaction with the national examination guidance method	0.159	1.66	0.101
Pressure of the national exam	0.078	0.997	0.322
Sense of learning good care	0.005	0.43	0.966
Sense of performing good care	−0.181	−1.784	0.078
Willingness to learn for good care	0.193	2.05	0.044
Confidence in my ability	0.246	2.504	0.014
Satisfaction with low back pain measure guidance	0.215	2.216	0.029
Japanese understanding of my own culture	0.003	0.037	0.97

Dependent variable; Intent to continue working. *P* value; Multiple regression analysis. Forced input method

Model summary: R = 0.749, R^2^ = 0.561, adjusted R^2^ = 0.485, standard error of estimates = 0.657

^†^: Reversal item: N1 ∼ N5 (Lower numerical value N1 is the highest level)

Note; The independent variables in Table 3 and Table 4 are the same except for “work engagement” and “well-being”.

We used the Mann-Whitney U test to analyze the difference between the 2 groups of Specific activities and Technical intern training, excluding Study abroad and Specific skills and Others, for which the sample size was small. The results showed that there was no significant difference between the 2 groups in subjective well-being (significance level [2-sided] 0.05). Furthermore, we attempted a comparative analysis in 3 countries, Indonesia, the Philippines, and Vietnam, except for a few nationalities whose sample sizes were extremely small (less than two). Since normality cannot be assumed for the data of these 3 countries and the sample size is small, the Kruskal-Wallis test was conducted. The results showed that there was no significant difference in work engagement and all its subscales among the 3 countries (significance level [2-sided] 0.05).

### Effect of various factors on the intent to continue working

**Fig. 1:**
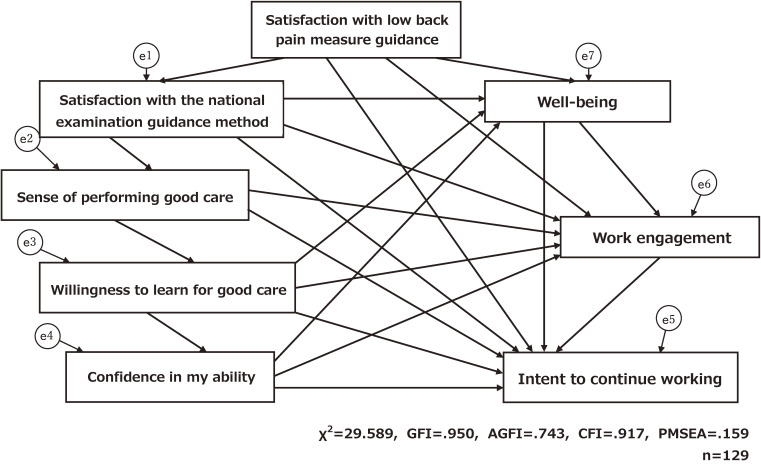
Effect of various factors related to intent to continue working: hypothetical model

A hypothetical model was set as shown in Fig. 1 for the effect with intent to continue working.

It consists of 7 variables that showed significant association with the intention to continue working as a result of multiple regression analysis with the intention to continue working as the dependent variable. The path (direction of the influence among variables) was constructed concerning the results obtained from the Spearman rank correlation analysis in this study. The goodness-of-fit index of this hypothetical model was goodness- of-fit index (GFI) = .950, adjusted goodness of fit index (AGFI) = .743, comparative fit index (CFI) = .917, root mean square error of approximation (RMSEA) = .159, and χ^2^ = 29.589.

Looking at the standardized coefficients, the direct effects on the intention to continue working were work engagement (β = .022, p = .005), well-being (β = .036, p = .029), confidence in my ability (β = .223, p = .013), satisfaction with low back pain measure guidance (β = .252, p = .001), and satisfaction with the national examination guidance method (β = .223, p = .002). There was no direct effect of motivation to learn on good care (β = .102, p = .370).

Toyoda [19] stated that the GFI and AGFI should be 0.9 or more and closer to 1.0, and the CFI, closer to 1.0, and the RMSEA, 0.05 or less. However, since the goodness of fit of the model should be considered relative to the purpose of the research [20], whilst improving the goodness of fit, the content validity from the viewpoint of educational support is not impaired. We thought that it would be necessary to give this sufficient consideration. Therefore, it was thought that the feeling of receiving effective instruction for low back pain would lead to satisfaction with the instruction for obtaining national certification as an achievement related to the practice and qualities of nursing care, and that the feeling of being able to execute good care would further motivate learning and create a sense of usefulness for oneself. Considering that this would lead to motivation, a pathway was set up that would lead from satisfaction with the instruction on how to prevent back pain to a sense of being able to provide good care and a desire to learn how to provide good care.

**Fig. 2:**
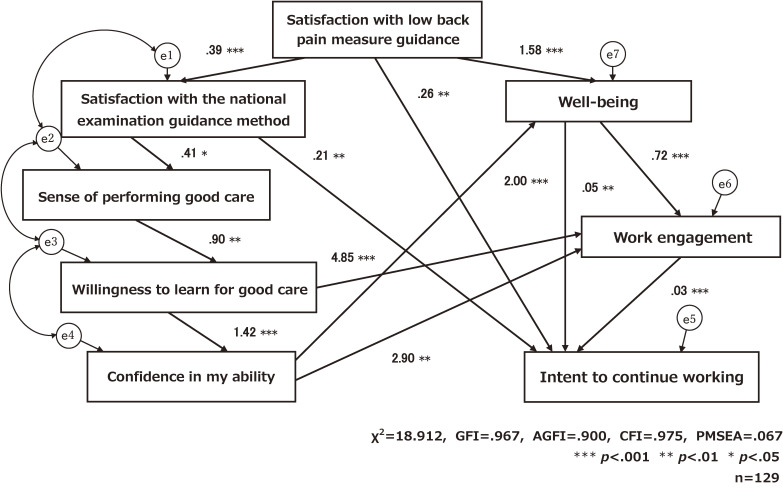
Effect of various factors on intent to continue working: modified model

Figure 2 shows the results of path analysis for this model. The goodness of fit indices for this model were GFI = .967, AGF = .900, CFI = .975, RMSEA = .067, and χ^2^ = 18.912, indicating a high degree of fit. Looking at the characteristics of each variable revealed by the model, the following direct effects were found for intent to continue working: satisfaction with low back pain measure guidance (β = .255, p < .002), satisfaction with the national examination guidance method (β = .217, p = .002), well-being (β = .046, p = .004), and work engagement (β = .026, p < .001). In addition, work engagement was found to have direct effects on well-being (β = .715, p < .001), willingness to learn good care (β = 4.849, p < .001), and confidence in my ability (β = 2.902, p = .005), whilst well-being was found to have direct effects on satisfaction with low back pain measure guidance (β = 1.582, p < .001) and confidence in my ability (β = 1.999, p < .001) had a direct effect on wellbeing.

### Reliability of the questionnaire

In the questionnaire used in this paper, the Cronbach α coefficient for the reliability of the original 20 items including the intent to continue working was 0.827.

## Discussion

The purpose of this study was to identify which of the original items contribute to “well-being” and “work engagement,” as well as to the “intent of foreign care workers to continue working.” Therefore, in the SEM, a hypothetical model was set and path analysis was performed concerning the Spearman rank correlation and multiple regression analysis results. The path analysis showed that “well-being,” “work engagement,” “satisfaction with low back pain countermeasure guidance,” and “confidence in my ability” were factors that directly affect the “intent to continue working” among foreign care workers. Factors that indirectly affect the “intent to continue working” were “sense of learning good care,” “sense of performing good care,” and “willingness to learn good care.”

From the goodness-of-fit index of the model, it is considered that a scientifically valid model with no discrepancy between the data and the model was generated.

In the multiple regression analysis in this study, “satisfaction with salary” did not show a significant effect on “intent to continue working,” showing the same trend as the report that wages alone cannot promote employee retention [21]. In addition, “willingness to learn good care” and “sense of performing good care,” which were found to affect “intent to continue working,” are theoretically consistent with poor quality of care for the elderly [7], inadequate technical guidance [22], and inappropriate education and training courses [23], which were cited as factors for turnover in previous studies. Although in a broad sense, we believe that the same trend was shown in this study. Furthermore, in the path analysis, “satisfaction with low back pain measure guidance” had a direct impact on “happiness” and “intent to continue working.” It is clear that low job satisfaction is strongly related to chronic low back pain that interferes with work [24]. In addition, when back pain that interferes with work persists for more than 3 months, subjective symptoms such as dizziness, stiff shoulders, and sleep disturbance are observed in the Occupational Stress Questionnaire, and psychological factors such as depression and anxiety scores tend to be higher [25]. Along with the results of these previous studies, the results of the current survey further support the idea that “satisfaction with low back pain measure guidance” is causally related to “well-being” and “intent to continue working.”

“The direct and indirect effects on “well-being” and “work engagement” were indicated by “satisfaction with low back pain measure guidance,” “satisfaction with the national examination guidance method,” “sense of performing good care,” “willingness to learn good care,” and “confidence in my ability.”

On the other hand, the Spearman rank correlation results showed that “salary satisfaction” and “Japanese language ability” were correlated with “intent to continue working,” but in the path analysis, no causal relationship was found with “intent to continue working.” Direct or indirect effects on “well-being” and “work engagement” were shown in the “sense of learning good care,” “willingness to learn good care,” and “confidence in my ability.” This means that acquiring high-quality care and utilizing individuals’ abilities in LTC work amplifies their well-being and that willingness to learn for quality care, rather than salary or Japanese proficiency, enhances the individuals’ work engagement in the intent of foreign care workers to continue working. It can be explained that it is not wages or Japanese language acquisition that brings pride, enthusiasm, and vitality in work. These findings differ from those of a report showing that a factor related to turnover is that foreign care workers think that it is enough if they can acquire high wages and Japanese ability [26] and of another report showing that the number of years of employment not accompanied by an increase in wages is a factor of job turnover in foreign care workers [27].

To increase the intent of foreign care workers to continue working, it is important to provide appropriate guidance for the development of back pain, to provide a setting and scene where good care can be learned, to utilize their abilities, and to provide educational support that motivates them to learn good care. In addition, to increase the motivation to learn good care, it is necessary to devise ways to take in more opportunities to feel that good care is being practiced. Employees in the high engagement group have been found to have higher levels of psychological well-being and personal accomplishment, whilst employees in the low engagement group had higher levels of emotional exhaustion and depersonalization [28]. This result was similar to the results in this study with different nationalities of participants. Furthermore, this study’s finding that “working period in Japan” was negatively correlated with “intent to continue working,” “work engagement,” and “well-being” suggests that facility managers should be attentive to the diverse needs of foreign workers as described in a previous study that explored the factors of better educational support methods for foreign LTC workers to continue working [18].

The limitations of this study are that the causal relationships between variables excluded from the hypothetical model during the SEM and latent factors other than the observed factors were not sufficiently analyzed this time. It is not possible to set up a hypothetical model using existing theoretically established factors/variables, and reliance on statistical analysis may have caused deviations in the results. Also, the multiple regression analysis result that “sense of performing good care” was negatively correlated with “intent to continue working” differed from the Spearman rank correlation and path analysis results. In multiple regression, the number of analysis targets ≥ 50 + 8 m is proposed with “m” as the explanatory factor [29, 30]. In this study, 202 sample sizes were required for 19 explanatory variables, and 129 were insufficient. Therefore, it can be said that overfitting may have occurred and the accuracy of prediction for unknown samples may have deteriorated.

However, these theoretical models/methods of educational support generated based on the findings of previous studies are useful in promoting the retention of foreign care workers involved in LTC based on work engagement and well-being. It is considered that the current results will contribute to solving the shortage of human resources in the field of LTC and welfare in Japan.

## Conclusion

To increase the motivation of foreign care workers to continue working, it is desirable to provide appropriate guidance related to the accumulation of back pain that is characteristic of LTC workers, and to create an environment where they can learn good care for national certification. It is effective to develop and maintain a relationship in practice that allows the individual to feel that his/her abilities are being utilized and educational support that motivates him/her to learn good care. And to increase the motivation to learn good care, it is necessary to incorporate many opportunities to feel that good care is being practiced.

## Data Availability

The datasets used and/or analyzed during the current study are available from the corresponding author on reasonable request.

## Abbreviations

LTC: Long-term care
J-SWBS: Japanese version of the Subjective well-being scale
J-UWES: Japanese version of the Utrecht work engagement Scale
SEM: Structural equation modeling
EPA: Economic partnership agreement
GFI: Goodness of fit index
AGFI: Adjusted goodness of fit index
CFI: Comparative fit index
RMSEA: Root mean square error of approximation
